# Acute Cerebellar Infarct in A Patient with Undiagnosed Fahr Syndrome: A Case Report

**DOI:** 10.5811/cpcem.20926

**Published:** 2024-10-14

**Authors:** R. Wesley Slaven, Martin Huecker, David Kersting

**Affiliations:** * University of Louisville, Department of Emergency Medicine, Louisville, Kentucky; † University of Louisville Health, Louisville, Kentucky

**Keywords:** Fahr syndrome, case report, posterior circulation stroke, cerebellar infarct

## Abstract

**Introduction:**

Fahr disease and Fahr syndrome represent clinical entities that result in diffuse intracranial brain calcification, either by way of genetic mutation in the case of the former or by secondary endocrine dysfunction in the latter.

**Case Report:**

We present a case of a middle-aged male with undiagnosed Fahr syndrome, identified during evaluation for symptoms of an acute posterior circulation cerebrovascular accident.

**Conclusion:**

Fahr syndrome is a clinical constellation of symptoms and radiographic findings often seen in late-stage hypoparathyroidism. The intracranial calcifications associated may be related to an increased risk for intracranial cerebrovascular disorders such as ischemic or hemorrhagic infarct.

## INTRODUCTION

Primary familial brain calcification, also known as Fahr disease, is a disorder characterized by calcification of the bilateral basal ganglia as well as a gradual-onset movement disorder. It is inherited in an autosomal dominant or sporadic fashion and usually presents in the fourth or fifth decade of life.^1^ Its manifestations typically involve clumsiness, gait disturbance, and involuntary movements or cramping. However, the constellation of symptoms can be variable in timing, severity, and character.^2^ Disease prevalence is noted at <1 per 1,000,000.^3^ Although calcifications primarily involve basal ganglia, thalamus, and subcortical white matter, the presence of symptoms varies greatly between patients.

Differentiating between Fahr disease and Fahr syndrome lies in the ability to identify secondary causes of brain calcification. Most commonly, underlying endocrine disorders, including hypoparathyroidism or hyperparathyroidism, represent the underlying cause of secondary Fahr syndrome.^4^ FLowenthal and Bruyn highlight that 21.5% of patients with idiopathic hypoparathyroidism develop Fahr syndrome,^5^ and multiple studies have demonstrated basal ganglia calcification following thyroidectomy.^6^,^7^ Case reports have shown a potential relationship between intracranial calcifications and cerebrovascular accidents, both hemorrhagic and ischemic;^8^,^9^ however, to our knowledge, no cases of cerebellar or posterior circulation infarcts have been described. In several reports, these events occur in patients with an otherwise favorable risk profile, including those of young age and without secondary risks of stroke.

## CASE REPORT

**Image 1:**
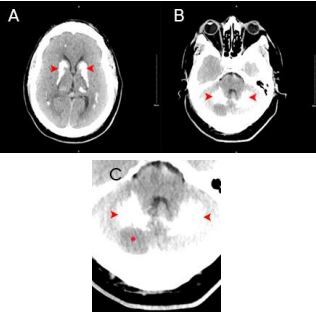
A) Non-contrast computed tomography of the head demonstrates diffuse calcification of the bilateral basal ganglia, thalami, and subcortical white matter of cerebral hemispheres (arrowheads). B) Calcification of the cerebellum bilaterally with area of hypodensity (arrowheads). C) Cerebellum with bilateral calcification (arrowheads) with area of hypodensity noted (asterisk).

**Image 2:**
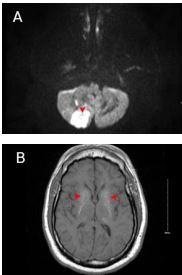
A) A magnetic resonance image of the brain shows an area of diffusion restriction in posterior-inferior right cerebellar hemisphere consistent with acute to subacute infarct, with susceptibility blooming (an artifact often seen surrounding prior hemorrhage or calcification, noted with arrowhead) along inferolateral margin consistent with petechial hemorrhagic transformation. B) Bilateral susceptibility blooming (arrowheads) in basal ganglia and cerebral white matter.

A 54-year old male with history of hypertension, diabetes, heart failure, and transient ischemic attack presented to the emergency department (ED) for several days of dizziness with a dull headache of moderate intensity. Symptoms began approximately three days prior to arrival, accompanied by blurry vision. Given his medical history and due to a concern for stroke or other intracranial pathology, a computed tomography (CT) of the head was obtained, which was notable for diffuse intracranial calcifications as well as a hypodensity in the posterior right cerebellar hemisphere (Image 1). Magnetic resonance imaging (MRI) showed an acute to subacute right posterior-inferior cerebellar artery stroke with early hemorrhagic transformation (Image 2). He was subsequently admitted to the neurology stroke service for further evaluation and management.

**CPC-EM Capsule**

What do we already know about this clinical entity?

*Fahr syndrome is a clinical syndrome present in late-stage hypoparathyroidism patients that may increase the risk of associated cerebrovascular accidents.*

What makes this presentation of disease reportable?

*This is the first demonstrated case in the literature of posterior circulation cerebrovascular accident in the Fahr syndrome population.*

What is the major learning point?

*The identification of radiographic findings suggestive of Fahr syndrome should prompt timely evaluation of secondary causes, namely endocrine disorders.*

How might this improve emergency medicine practice?

*We highlight a rare sequela of endocrine disorder that warrants careful optimization, often with a multidisciplinary team.*

Additional history obtained following admission showed that this patient reported a previous history of thyroid carcinoma with subsequent resection at age six, not disclosed at the time of his initial encounter with ED staff, with resultant post-procedural hypothyroidism and hypoparathyroidism. He also reported multiple bouts with ureterolithiasis bilaterally, and cataracts that were to be intervened upon at a later date. Following admission, angiogram studies revealed right vertebral artery occlusion with reconstitution, as well as severe left vertebral artery stenosis. The patient subsequently underwent vertebral artery stenting and risk factor management including dual antiplatelet therapy and initiation of a high-intensity statin. He was discharged four days following admission to outpatient follow-up.

Following discharge, the patient was seen in neurology clinic where his National Institutes of Health Stroke Scale score was zero. He did report issues with forgetfulness, but otherwise had no residual neurologic deficit. In addition, he has had multiple admissions for treatment of symptomatic nephrolithiasis, requiring multiple ureteral stents and lithotripsy procedures. To date, he is doing well in regard to his neurologic status and is currently followed on an outpatient basis by neurology, as well as by nephrology, otolaryngology, and cardiology.

## DISCUSSION

Fahr disease is a rare cause of neurologic symptoms of variable severity and quality. Idiopathic calcifications most commonly affect the basal ganglia, thalamus, and cerebral white matter.^10^ Computed tomography remains a reliable initial test for evaluation, given that these calcium deposits are hyperdense and their locations and abundance often point toward the distinct disease entity of Fahr disease/syndrome. MRI findings can vary depending on the composition of deposits and stage of disease. As a result, MRI may exhibit a near-normal appearance in a patient whose CT imaging is grossly abnormal.^11^

While Fahr disease represents a distinct, primary pathology leading to the finding of basal ganglia calcification, the disease prevalence is less than one per million. Highlighting this rarity, an evaluation into secondary causes of these diffuse calcifications is warranted, with special attention paid to endocrine disorders as an underlying driving force.

In a 1981 report by Harrington et al, 42 of 7,000 patients were retrospectively identified as having basal ganglia calcifications on CT of the head, an incidence of 0.6%. Of these patients, 26 were available for follow-up, and two of the 26, or 7.7%, were found to have evidence of parathyroid dysfunction.^12^ Emergency physicians might encounter this disease process over the course of their career. Recognition of this clinical entity is of paramount importance, as these patients warrant prompt evaluation for underlying causes, such as endocrine disorders. While this may not take place in an emergency setting, subspecialist referral is necessary to ensure appropriate long-term symptom management.

In this patient, his initial presentation and workup focused on headache and dizziness. While the spectrum of symptoms in Fahr syndrome is broad, most patients do not experience headaches or dizziness. However, this may be confounded in that patients may experience gait disturbance and Parkinsonian symptomatology,^13^ which may mimic sensations of dizziness or, alternatively, limit history. Unfortunately, he had a concurrent acute pathology in the form of an acute cerebellar stroke contributing to his symptoms. While previous case reports have described both hemorrhagic and ischemic cerebrovascular accidents in patients found to have radiographic evidence of Fahr syndrome/disease, none have shown presence of a posterior circulation stroke.

Regardless, in this patient it remains a possibility that his underlying endocrine disorder played a role in the development of his cerebrovascular accident and, potentially, his prior transient ischemic attack. Unbeknownst to ED staff at the time, this patient represented a classic array of symptomatic hypoparathyroidism, with ureterolithiasis and resultant kidney disease, coronary artery disease, hypothyroidism, and Fahr syndrome, diagnosed during his initial visit. How this syndrome translates to clinically significant cerebrovascular conditions is a subject of speculation. It has been shown that calcium deposits into the walls of arterioles, capillaries, and veins.^14^ Additionally, a postmortem examination of a patient with Fahr disease published in 2004 shows extensive calcifications of small blood vessel walls on the dentate nucleus of the cerebellum. Inflammatory changes and deposition in endothelial and stromal vascular cells were also observed.^15^ The combination of intravascular calcium deposition and associated inflammation may increase risk of regional ischemia, aneurysm formation, or associated hemorrhagic insult.

## CONCLUSION

We present a patient with a phenotypic presentation of symptomatic, idiopathic hypoparathyroidism with resultant development of diffuse intracranial calcification, known as Fahr syndrome. This constellation was identified during evaluation for a concurrent, acute right posterior inferior cerebellar artery infarct.
